# Possible healthcare-associated transmission as a cause of secondary infection and population structure of *Staphylococcus aureus* isolates from two wound treatment centres in Ghana

**DOI:** 10.1016/j.nmni.2016.07.001

**Published:** 2016-07-12

**Authors:** G. Kpeli, I. Darko Otchere, A. Lamelas, A.L. Buultjens, D. Bulach, S.L. Baines, T. Seemann, S. Giulieri, Z. Nakobu, S.Y. Aboagye, E. Owusu-Mireku, G. Pluschke, T.P. Stinear, D. Yeboah-Manu

**Affiliations:** 1) Noguchi Memorial Institute for Medical Research, Accra, Ghana; 2) Swiss Tropical and Public Health Institute, Basel, Switzerland; 3) University of Basel, Basel, Switzerland; 4) Doherty Applied Microbial Genomics, Department of Microbiology and Immunology, Doherty Institute for Infection and Immunity, University of Melbourne, Melbourne, VIC, Australia; 5) Victorian Life Sciences Computation Initiative, University of Melbourne, Parkville, VIC, Australia; 6) Red de Estudios Moleculares Avanzados, Instituto de Ecología, A.C, Carretera antigua a Coatepec 351, El Haya Xalapa, Veracruz, Mexico

**Keywords:** Buruli ulcer, health care-associated, infection, spa typing, *Staphylococcus aureus*, whole genome sequencing

## Abstract

We have previously shown that secondary infections of Buruli ulcer wounds were frequently caused by *Staphylococcus aureus.* To gain understanding into possible routes of secondary infection, we characterized *S. aureus* isolates from patient lesions and surrounding environments across two Ghanaian health centres. One hundred and one *S. aureus* isolates were isolated from wounds (*n* = 93, 92.1%) and the hospital environment (*n* = 8, 7.9%) and characterized by the *spa* gene, *mecA* and the Panton–Valentine leucocidin toxin followed by *spa* sequencing and whole genome sequencing of a subset of 49 isolates. *Spa* typing and sequencing of the *spa* gene from 91 isolates identified 29 different *spa* types with t355 (ST152), t186 (ST88), and t346 dominating. Although many distinct strains were isolated from both health centres, genotype clustering was identified within centres. In addition, we identified a cluster consisting of isolates from a healthcare worker, patients dressed that same day and forceps used for dressing, pointing to possible healthcare-associated transmission. These clusters were confirmed by phylogenomic analysis. Twenty-four (22.8%) isolates were identified as methicillin-resistant *S. aureus* and *lukFS* genes encoding Panton–Valentine leucocidin were identified in 67 (63.8%) of the isolates. Phenotype screening showed widespread resistance to tetracycline, erythromycin, rifampicin, amikacin and streptomycin. Genomics confirmed the widespread presence of antibiotic resistance genes to β-lactams, chloramphenicol, trimethoprim, quinolone, streptomycin and tetracycline. Our findings indicate that the healthcare environment probably contributes to the superinfection of Buruli ulcer wounds and calls for improved training in wound management and infection control techniques.

## Introduction

Microbial contamination and colonization of wounds is common to all wounds healing by secondary intention and has been proposed as a precondition to the formation of granulation tissue and stimulation of wound healing [1]. Wounds can be infected through three main sources; the surrounding skin, endogenous sources such as the nasal mucosa, gastrointestinal tract and genitourinary tract and the wider environment. Within a healthcare facility, the sources of contamination and subsequent infection of a wound may include healthcare workers (HCW), patients and the inanimate environment. Direct contact of a patient with an infected HCW during general care or medical treatment can result in the transmission of microorganisms. Indirectly, an infected patient or HCW could touch and contaminate an object, instrument or surface and subsequent contact between the item and a second patient is likely to contaminate that patient leading to infection. Wound microflora is usually polymicrobial [2] comprising organisms such as staphylococci, enterococci, streptococci, facultative Gram-negative bacteria and anaerobic bacteria [3].

*Staphylococcus aureus* is a notorious opportunistic nosocomial pathogen, and one of the main organisms involved in the infection of chronic wounds [4]. It may be carried asymptomatically by a carrier and transferred from patient to patient. It is estimated that about 25% of the normal population may be carriers, with higher carriage rates around 50% in patients with insulin-dependent diabetes, intravenous drug users and patients on dialysis [5].

In a previous study, we identified infection of Buruli ulcer (BU) wounds by bacterial pathogens as a possible cause of wound healing delay among study participants [6]. Several bacterial species were isolated with *S. aureus* and *Pseudomonas aeruginosa* dominating. Despite the finding that most of the organisms identified were nosocomial pathogens, the possible routes of infection of the lesions were not examined in this study. In seeking to have a better understanding of the possible routes of infection of the lesions, we initiated the current study to characterize *S. aureus* isolated from different sources in two health facilities in Ghana treating BU.

## Materials and methods

### Study sites, participants and sample collection

The study was carried out at the two main BU treatment centres in the Ga West and Ga South districts of the Greater Accra Region of Ghana, designated facility A and facility B, respectively. The study involved the analysis of *S. aureus* isolates from wounds of microbiologically confirmed BU (BU) and non-BU (NBU) patients (initially suspected BU cases that were not confirmed by any of the three confirmation methods—Ziehl–Neelson microscopy, IS2404 PCR and culture) who received treatment either as inpatients on admission or outpatients at one of the two health centres from October 2010 to February 2014. Lesions of patients were swabbed and the samples were analysed at the Bacteriology Department of the Noguchi Memorial Institute for Medical Research. A total of 173 samples were collected from 162 patients and *S. aureus* was isolated from 88 samples originating from 76 patients, 61 of whom were BU patients and 15 were NBU patients. Of the 61 BU patients, 56 were outpatients and five were inpatients at the time of sampling, whereas all the NBU patients were outpatients. The five inpatients had been on admission for 4 weeks or more and therefore isolates from these patients could be classified as potentially hospital acquired. To elucidate the sources of infection, between August 2013 and February 2014 the hands of HCW, dressing rooms and wards of the health centres, surfaces of dressing tables, door handles, instruments and equipment as well as dressing solutions and materials were sampled for microbiological analysis. Swab samples were also taken from the palms of nurses and their gloved hands in between dressing of patients and the lesions of the patients they dressed before and after dressing. Swab samples were transported in phosphate buffered saline at 4°C to the Noguchi Memorial Institute for Medical Research. Eighty-six samples were collected and analysed and *S. aureus* was isolated from 13 of them (see Supplementary material, Fig. S1).

### Microbiological methods

The samples were processed and inoculated on blood and mannitol salt agars and incubated for 18–24 h at 37°C, after which they were examined. Identification of *Staphylococcus* species was by colony and microscopic morphology, catalase reaction and the coagulase biochemical test (BD, Franklin Lakes, NJ, USA). The Staphylase Kit, BD BBL™ Staphyloslide Latex Test (BD, Franklin Lakes, NJ, USA) was used to differentiate the catalase-positive Gram-positive bacteria *S. aureus* from other *Staphylococcus* species.

### Antibiogram of isolated bacteria

Antibiotic susceptibility testing was determined by the Kirby–Bauer disc diffusion method according to CLSI guidelines [7]. Susceptibility was determined for the antibiotics; amikacin (30 μg), sulphamethoxazole-trimethoprim (23.75 μg/1.25 μg), ampicillin (10 μg), tetracycline (30 μg), gentamicin (10 μg), erythromycin (15 μg), cefuroxime (30 μg), ceftriaxone (30 μg), chloramphenicol (30 μg), cefoxitin (30 μg), rifampicin (5 μg), streptomycin (10 μg), vancomycin (30 μg), clindamycin (2 μg) and cefotaxime (30 μg). Cartridges of antibiotics were obtained from Oxoid (Wade Road, Basingstoke, Hampshire, United Kingdom) and BD. *Staphylococcus aureus* ATCC 25923 was used as reference strain.

### Genotyping—traditional molecular typing

Crude DNA extracts were prepared by boiling and used as template in the PCR. Genetic relatedness of the isolates was determined through amplification of the polymorphic X region of the protein A (*spa*) gene using the primer pairs spa-1113F and spa-1514R (see Supplementary material, Table S1) [8] and DNA from *S. aureus* ATCC^®^ 25923 as positive control. A total of 95 isolates out of the 101 were typed using these primers. A number of suspected *S. aureus* strains could not be typed by these primers and were further typed using another set of primers, spaT3-F and spa-1517R (see Supplementary material, Table S1). These primers were previously described to detect *S. aureus* strains that may have rearrangements in the IgG-binding region of the gene where the forward primer is located, making them untypeable by the original *spa* primers [9]. Sequencing of the *spa* gene from positive isolates was outsourced to Macrogen Europe, (Amsterdam, the Netherlands) and sequences were analysed and assigned to *spa* types using DNAGear [10]. The relationship between strains was investigated using the Staden package [11] and a maximum likelihood phylogenetic tree produced in Mega 5.05 (www.megasoft.net) and visualized in FigTree v1.4.2. (http://tree.bio.ed.ac.uk/software/figtree/).

Genes for *mecA* and Panton–Valentine leucocidin (PVL) were sought using the primers mecA P4, mecA P7 and pvl-F, pvl-R (see Supplementary material, Table S1) [12], [13]. A mecA and PVL-positive isolate *S. aureus* 282-101 from the Statens Serum Institute (Copenhagen, Denmark) kindly provided by Dr Beverly Egyir, was used as positive control. Agr types were determined using the primers pan agr F, agr 1-R, agr 2-R, agr 3-R and agr 4-R (see Supplementary material, Table S1) [14]. The PCR contained 12.5 μL HotStar*Taq* Mastermix (Qiagen, Hilden, Germany), 5 μL nuclease-free water, 2 μL of each primer and 3.5 μL of DNA. Results of *mecA*, *agr* and PVL typing were analysed using Microsoft Excel.

### Whole genome sequencing and analysis

From the 101 isolates, a subset of 70 isolates were selected for whole genome sequencing (WGS) made up of 31 methicillin-resistant *S. aureus* (MRSA) isolates and 39 other methicillin-susceptible *S. aureus* (MSSA) isolates spanning the clusters observed from the phylogenetic tree obtained from sequencing of the *spa* gene. Genomic DNA was extracted with the Qiagen DNeasy Blood and tissue kit (Qiagen) according to the manufacturer’s instructions and sequenced using the Illumina MiSeq platform (2× 250 bp sequencing by synthesis chemistry using DNA libraries prepared using Nextera XT (Illumina, San Diego, CA, USA). Resulting sequence reads were processed using a custom bioinformatics analysis pipeline Nullarbor (nullarbor.pl 0.6; https://github.com/tseemann/nullabor) to *de novo* assemble and also align read data against the Sa_aus0325 reference genome (S. Giulieri *et al.*, unpublished). Nullarbor uses a BLAST-based method to screen contigs from *de novo* assemblies for the presence of antibiotic resistance genes. Pairwise alignments of core genome single nucleotide polymorphisms (SNPs) were used with FastTree [15] to infer maximum likelihood phylogenetic trees using the general time reversible model of nucleotide substitution. Resulting trees were visualized in FigTree v1.4.2. The topologies of rooted phylogenetic trees obtained from both *spa* typing and WGS using *Staphylococcus simiae* CCM7213 as an outgroup were compared by tanglegram in Dendroscope (v3.4.4) [16].

## Results

### Bacterial isolates

In all, 101 *S. aureus* isolates were identified from the sources sampled. Eighty-eight isolates were recovered from 76 patients during routine sampling; 72 (81.6%) were from 61 BU patients and 15 were from NBU wounds. One isolate each was identified from 66 patients and multiple isolates from the other ten patients. Five patients had two isolates from a single lesion collected at one time-point, one patient had two isolates from two lesions, three isolates were identified from a patient with three lesions (patient A, one from each), two patients had two isolates sampled at two different time-points during the course of treatment, and one patient with three isolates sampled thrice at different time-points during the course of treatment.

To understand possible route of infection, additional sampling of HCW, working environment and patients being attended to led to isolation of 13 (15.1%) *S. aureus*. The isolates comprised five from patient lesions, six from equipment (forceps) and one each from the hand of an HCW and a table used for dressing wounds. All of these isolates were from Facility B. In total, 53 isolates were from Facility A and 48 isolates were from Facility B.

### Population structure of *S. aureus* and epidemiological association

**Fig. 1:**
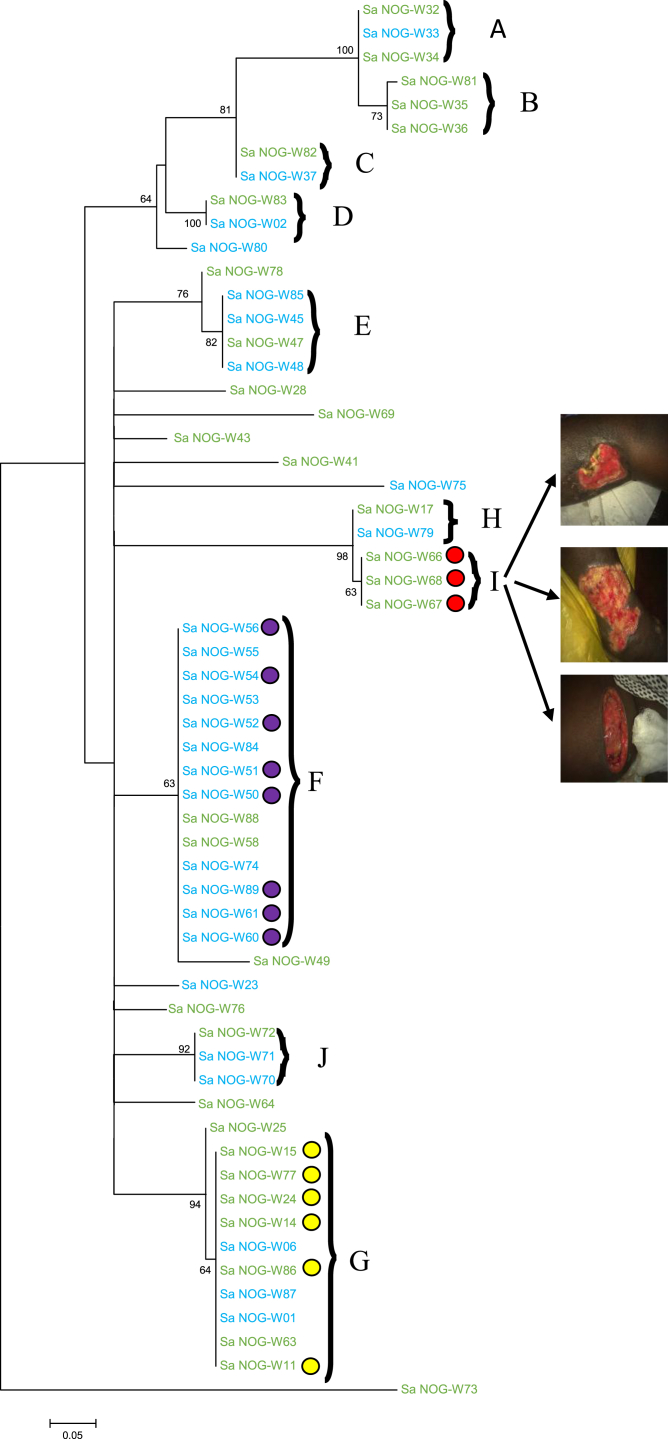
*Spa* phylogeny showing clusters and relationships between isolates. Maximum likelihood phylogeny of *spa* gene. The tree was rooted in the midpoint. Numbers in nodes indicate support values in the form of proportions of bootstrap pseudoreplicates. Branches with support values >55% are collapsed. A–J = the clusters identified. The green-coloured strains are from Health centre A, and the blue ones are from Health centre B. The yellow-coloured circles represent methicillin-resistant *Staphylococcus aureus* and the violet circles show isolates from a healthcare worker, patients and equipment in Health centre B.

*Spa* typing identified 29 different *spa* types including 15 (14.9%) singles. The three dominant *spa* types (t355, t186 and t346) were found in seven (6.93%) isolates each (see Supplementary material, Table S2). Thirty-five isolates could not be assigned a *spa* type. Phylogenetic analysis identified ten different clusters, where a cluster was defined as a group of two or more isolates found on the same branch of the tree (Clusters A–J; Fig. 1), which consisted of health-centre-specific clusters and common clusters. Clusters A, C, D, H and J were made up of strains from both health centres (Fig. 1) with rates of 2:1, 1:1, 1:1, 1:1 and 1:2 for Facilities A and B, respectively. Clusters B and I were made up of strains from Facility A only, whereas seven out of the ten isolates in cluster G were also from Facility A. Clusters E and F were made up mainly of strains from Facility B with rates of 1:3 and 2:12 for Facility A and B, respectively (Fig. 1). Within cluster F were isolates cultured from samples taken on the same day from the hand of an HCW and patients dressed consecutively by this worker, isolates from samples taken on a different day from one equipment (forceps) after it had been used on four patients consecutively and two isolates recovered from a patient at two different time-points (weeks 2 and 8) during treatment (Fig. 1). This finding led us to conclude that transmission events were ongoing within this health centre. Cluster G was made up mainly of MRSA isolates (Fig. 1) whereas cluster I was made up of isolates from three different lesions of one patient that had the same *spa* type (t2500) (Fig. 1),, suggesting that the three lesions were infected from a common source, possibly from the patients’ microbiota.

### Detection of *mecA* and PVL genes and Agr type

The PCR screening for the *mecA* gene identified 31 isolates as MRSA. The genotypes of 24 of these isolates correlated with their antibiogram results; however, *mecA* was also detected in seven isolates showing susceptible phenotypes. Twenty-one of these were from BU patients, eight were from NBU patients and two were from the environment. Four of the MRSA were isolated from inpatients. The PVL gene was detected in 66 isolates; 51 from BU patients, 12 from NBU patients and three from the environment. Both *mecA* and the PVL genes were detected in 20 isolates made up of 14 from BU patients, five from NBU patients and one from the environment. Nineteen (18.8%) isolates belonged to agr type 1, 23 (22.8% ) isolates to agr type 2, 30 (29.7%) isolates to agr type 3, three (2.9%) isolates to agr type 4, and four (3.9%) isolates showed bands for both agr types 2 and 3. Twenty-two (21.8%) isolates were non-typeable and possibly agr-defective mutants.

### Antibiogram of isolates

Over 70% of the isolates were susceptible to amikacin (89; 88%) and gentamicin (89; 88%). Resistance rates >50% were recorded against the antibiotics ampicillin (91; 90%), tetracycline (57; 56.4%) and chloramphenicol (67; 66%). Lower resistance rates of 37.6%, 39.6% and 48.5% were also recorded against the cephalosporins; ceftriaxone, cefotaxime and cefuroxime, respectively, whereas 26% of the isolates showed reduced susceptibility to vancomycin (Table 1). Comparing the antibiograms of MRSA and MSSA isolates, a significant proportion of MRSA isolates were resistant to the tetracycline (p <0.05) (Table 2). Resistance to chloramphenicol was equally high among MRSA and MSSA.

### WGS analysis

During the course of this project we had the opportunity to investigate some of the isolates further by WGS. From an initial 70 isolates subjected to WGS, 21 were of low read-coverage and excluded from analysis. We first inferred multilocus sequence typing data from the WGS data. The 49 isolates belonged to 12 different sequence types (ST) with ST15 (13 isolates) and ST88 (11 isolates) dominating. The remainder belonged to ST1 (one isolate), ST5 (six isolates), ST6 (one isolate), ST72 (two isolates), ST121 (two isolates), ST152 (three isolates), ST395 (one isolate), ST707 (one isolate), ST2434 (one isolate) and ST3248 (three isolates) (see Supplementary material, Data S1). Four isolates represented new STs. The 11 ST88 isolates were all MRSA and will be described elsewhere (Kpeli *et al.*, manuscript in preparation).

**Fig. 2:**
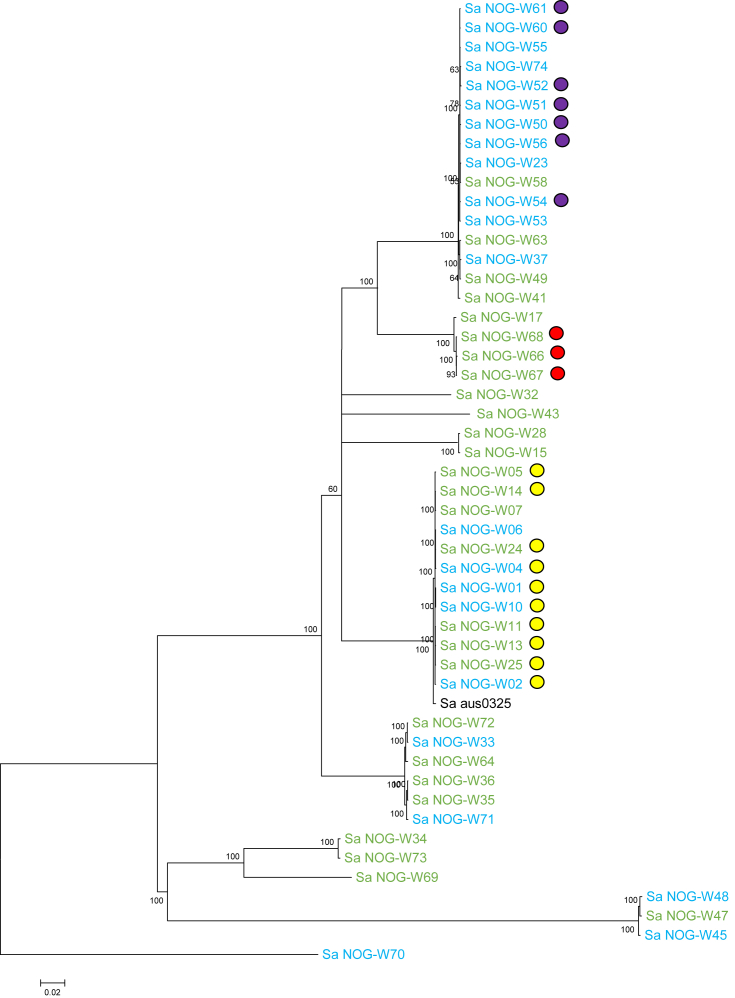
Whole genome phylogeny of sequenced isolates. Maximum likelihood phylogeny of whole genome sequencing isolates. The tree was rooted in the midpoint. Numbers in nodes indicate support values in the form of proportions of bootstrap pseudoreplicates. Branches with support values >55% are collapsed. Green-coloured strains are from Health centre A and the blue ones are from Health centre B. The red-coloured circles represent isolates from patient A, yellow-coloured circles represent methicillin-resistant *Staphylococcus aureus* and the violet circles show isolates from a healthcare worker, patients and equipment in Health centre B.

Read-mapping of the 49 genomes against the Sa_aus0325 reference sequence produced a 2.2-Mbp core genome with 100 361 SNPs. A maximum likelihood phylogeny was inferred from pairwise comparisons of these SNPs (Fig. 2). Among the 13 ST15 isolates, ten were isolated from one health centre, including from the hand of an HCW, patients and equipment, corresponding to results from *spa* typing and giving support to the conclusion that transmission events were ongoing within this health centre. The three isolates from patient A were of the same ST type (ST3248) and between them had SNP differences of 29 bp, 51 bp and 34 bp (see Supplementary material, Data S2) also corresponding with the *spa* typing results discussed above. This small number of SNP differences points to the isolates spreading from a common source.

We then inferred the resistome of each isolate from the WGS data (see Materials and methods). Antibiotic resistance genes coding for resistance to β-lactams (*blaZ*), chloramphenicol (*cat* and *catpC221*), trimethoprim (*dfrG*), methicillin (*mecA*), quinolone (*norA*), streptomycin (*str*) and tetracycline (*tetK*, *tetL* and *tetM*), were identified in 48 (98%), one (2%), 22 (45%), 13 (25.5%), 13 (25.5%), 49 (100%), 12 (24.5%), eight (16.3%), 12 (24.5%) and 12 (24.5%) of the 49 isolates, respectively (see Supplementary material, Data S1). We further investigated the *rpoB* gene of rifampicin-resistant strains and identified two known amino acid substitutions H481N and I527M implicated in rifampicin resistance in seven and one isolate, respectively (see Supplementary material, Data S1). Other mutations were also found within the rifampin resistance-determining (Rif) region of the rifampicin-resistant isolates but further studies are needed to ascertain if these mutations contribute to rifampicin resistance.

**Fig. 3:**
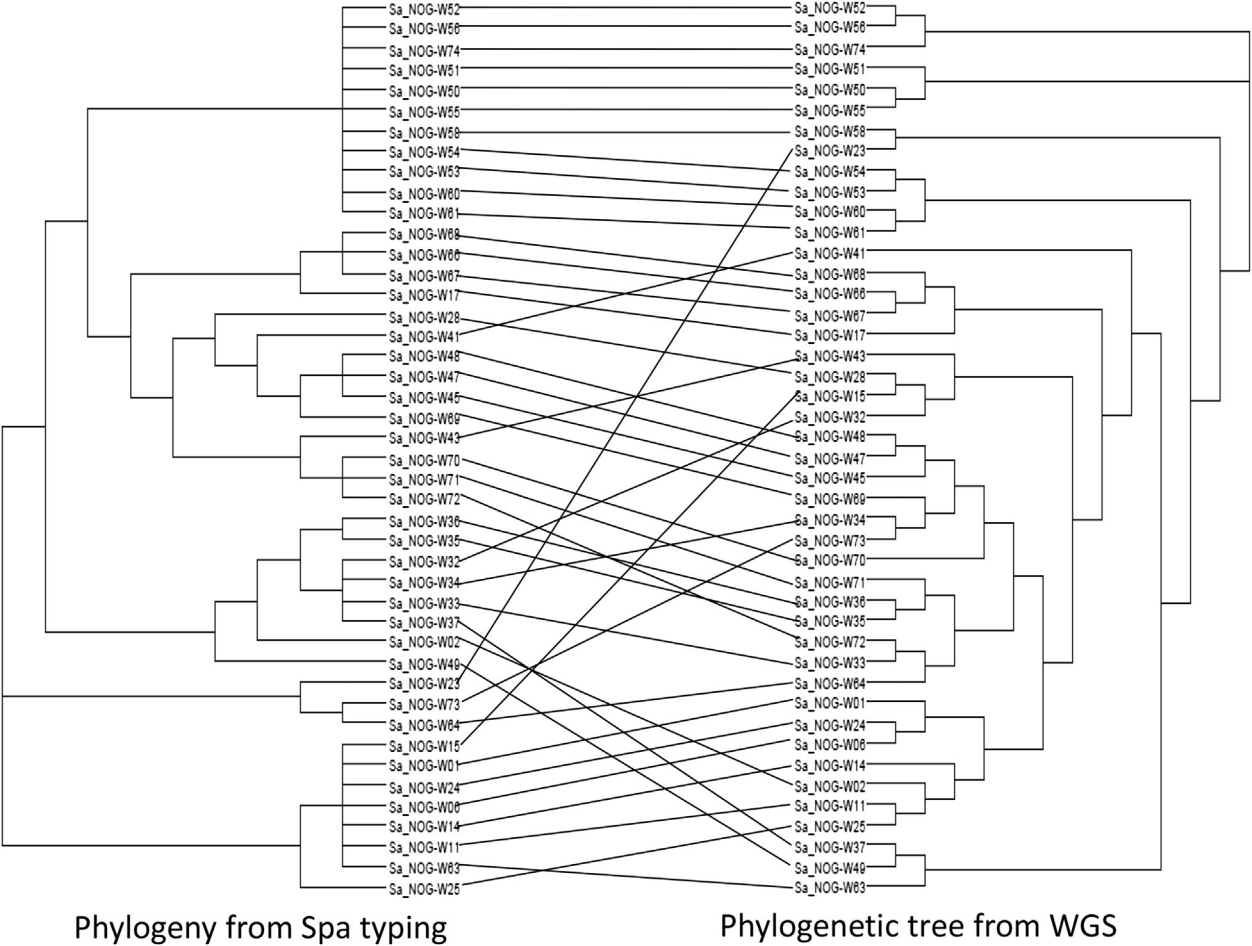
Tanglegram of *spa* and whole genome phylogenies. Tanglegram of *spa* (a) and whole genome sequencing (b) phylogenies produced in dendroscope. Topologies show some agreement between phylogenies but an overall lack of congruence.

Analysis of the topologies of the phylogenies produced by *spa* typing and WGS shows lots of agreement but also some differences (Fig. 3). Clustering of MRSA isolates from patient A and isolates from facility B from an HCW, the patients dressed by this worker and equipment were both predicted by the two methods. However, though *spa* typing predicted the clustering of isolate SA_NOG-W15, which is an MRSA, with other MRSA isolates, this cluster was not confirmed by WGS, which predicted it to cluster with isolate SA_NOG-W28, an MSSA.

## Discussion

This study confirmed healthcare-associated infection (HAI) as a source of wound infection within our study health centres. Our analysis shows that *spa* typing is useful for predicting transmission patterns in resource-limited settings but that there is also a need for access to low-cost microbial genomics in developing countries. Genome analysis rapidly revealed widespread antibiotic resistance among the isolates and clearly identified likely transmission clusters.

In previous work [6], we found that wound infection may be a source of healing delay. The findings from our current study implicate the healthcare environment (including healthcare personnel) as possible sources of *S. aureus* infection. From our cluster analysis using *spa* typing, we inferred three modes of wound infection; two health-facility-related sources through an HCW and the inanimate environment (Fig. 1), and the third source through self infection (Fig. 1). This result corresponds with that of previous studies, which have implicated HCW, patients and the inanimate environment in the transmission and subsequent acquisition of *S. aureus* in healthcare settings [17]. HAIs are known to negatively impact healthcare delivery around the world. Effective infection prevention and control practices, especially compliance with hand hygiene recommendations, will lead to significant reduction in the rate of HAIs. Ghana has a policy document to aid the training of HCW in infection prevention and control (www.ghanahealthservice.org/division-scat.php?ghsdid=6&ghsscid=96). However, a monitoring survey in selected health facilities within the Greater Accra region showed that the compliance level of HCW to these guidelines is low (Ghana Health Service 2011, *Infection Prevention and Control. A survey in Greater Accra*, unpublished) with rates below the 70% recommended by the WHO. Adherence to strict policies supported by periodic training and monitoring of HCWs is required to ensure compliance with existing infection prevention and control guidelines and to decrease the frequency of HAIs.

With regard to the patient-specific clusters, Yeboah-Manu *et al.* [6] reported that some patients recycle bandages used in wound dressing because of inadequate financial support during their treatment period, and this could result in the transfer of pathogens from one lesion to another. Additionally, wound management in Ghana is influenced to a high degree by local traditional beliefs and practices. Beliefs revolving around the category of people deemed qualified to manage wounds affect the behaviour of patients. A recent study from our team (Koka *et al.* manuscript in preparation) reports that in many communities, pregnant women and nursing mothers are seen as unqualified to manage wounds and in cases where HCWs fall into this category, patients resort to the re-dressing of their wounds after the HCW has dressed them. This could also lead to the transfer of pathogenic organisms into the lesions as patients do not observe proper aseptic procedures during wound redressing. Hence, there is a need to counsel patients to adhere to the biomedical wound care and management practices to reduce or avoid self infection of their lesions.

This study was limited by not performing a thorough investigation of other body sites of the patients where *S. aureus* is known to exist as a normal flora. An exhaustive investigation should have included culture of samples from other areas of the patients such as the skin, hand and anterior nares of the nasal cavity to compare between strains from these sites and the wounds. The study may also have been affected by the Hawthorne effect at Facility A and this could account for no *S. aureus* isolates from sampling the environment and HCWs at this facility.

Two methods were employed to arrive at our conclusion of HAIs; the single gene locus DNA sequence-based marker *spa* typing and WGS. As revealed from our analysis, phylogenies from both methods predicted similar clusters. Whereas WGS reveals variability across the whole genome and is able to discriminate down to single nucleotide differences, *spa* typing looks at genetic variability at a single locus between 200 and 600 bp in length. *Spa* typing is less expensive and demanding in terms of infrastructure and expertise and has a shorter turnaround time compared with WGS. Our results show that the level of discrimination of *spa* typing is adequate to guide infection control and also supports its use in epidemiological studies. However, the lack of congruence between the two methods probably indicates that spa typing lacks resolution to be able to differentiate between highly genetically related isolates. This partial sequencing technique also cannot reveal the finer genetic details that accumulate during the evolution of bacterial populations. Therefore, although *spa* typing is useful for the prediction of transmission events in resource-limited settings, where access to the newer, more expensive and more advanced WGS technologies is limited, we should be looking for ways to implement low-cost microbial genomics in these countries because the rich data obtained from pathogen genomes can be used to make well-informed decisions to control the spread of disease.

Another important finding from this study is the high genetic diversity and PVL-positivity among the isolates. This agrees with existing knowledge on the genetic diversity and PVL prevalence among African *S. aureus* isolates [18], [19], [20]. It also supports the assertion that Africa is a PVL endemic continent with high prevalence of PVL being a distinguishing trait of African *S. aureus* isolates [19] compared with Asia, Europe and the USA [21]. Additionally, we identified t355 as one of the most prevalent *spa* types. This correlates with studies in Ghana and other African countries that identified this *spa* type as one of the most widespread, hence suggesting it to be widely established and distributed in Africa [18], [22], [23].

Clinical *S. aureus* isolates are known to be *agr* positive [24] and this is also evidenced from our results with 79 (78.2%) isolates having this locus. However, 22 (21.8%) isolates were defective for *agr* function. Previous research suggests that *agr*-defective mutants can interact with *agr*-positive variants during clinical infection [24]. These *agr*-defective mutants play an important role in persistent infection by forming thicker biofilms compared with *agr*-positive isolates [25]. This phenomenon might play a role in delayed wound healing, which we observed among patients within our study health centres [6].

Antibiotic resistance rates from both phenotypic and resistome investigations confirm the increasing prevalence of drug resistance in Ghana [26]. Thirty-one isolates (30.7%) were confirmed as MRSA. These data are consistent with the recognized fact that Africa has an intermediate prevalence of MRSA, usually between 25% and 50% [27]. Of the 31 isolates, seven showed susceptible phenotypes, though the *mecA* gene was detected in molecular analysis. Phenotypically susceptible *mecA*-positive clones have been reported by various research groups [28], [29]. The MRSA phenotype is regulated by two genes *mecI-mecR1.* The induction of MRSA expression by this system is, however, very slow and may render some isolates with the *mecA* gene phenotypically susceptible. The existence of such strains represents a hidden reservoir for transmission of the methicillin-resistance gene in any environment. In many resource-limited settings, clinicians mostly rely on the results of culture and drug susceptibility testing to guide treatment of patients and most laboratories are also not equipped for molecular testing of organisms. As these clones can only be detected through molecular analysis, they will be reported as susceptible organisms, which will lead to treatment failure.

Of equal concern is the observed high vancomycin resistance of 26%, which is comparable to vancomycin-resistant rates of between 57.7% [30] and 89% [31] that have been reported by other studies from West Africa.

## Conclusion

Our findings indicate that healthcare-associated transmission contributes to wound infection and call for periodic training in infection prevention and control practices to prevent the occurrence of epidemics of nosocomial MRSA.

## Figures and Tables

**Table 1:** Antibiogram of isolates

Antibiotics	Antibiogram		
Sensitive, *n* (%)	Intermediate, *n* (%)	Resistant, *n* (%)
Amikacin	89 (88)	7 (7)	5 (5)
Cefotaxime	50 (49.5)	11 (10.8)	40 (39.6)
Gentamicin	89 (88)	4 (4)	8 (8)
Tetracycline	39 (38.6)	5 (5)	57 (56.4)
Chloramphenicol	26 (26)	8 (8)	67 (66)
Ceftriaxone	53 (52.5)	10 (10)	38 (37.6)
Cotrimoxazole	65 (64.3)	4 (4)	32 (31.6)
Cefuroxime	52 (51.5)	0	49 (48.5)
Ampicillin	6 (6)	4 (4)	91 (90)
Clindamycin	52 (51.4)	18 (18)	31 (30.6)
Cefoxitin	66 (65)	5 (5)	30 (30)
Erythromycin	41 (41)	37 (36)	23 (23)
Rifampicin	46 (45.5)	9 (9)	46 (45.5)
Streptomycin	66 (65.3)	9 (9)	26 (25.7)
Vancomycin	75 (74)		26 (26)

**Table 2:** Comparison of antibiotic resistance between methicillin-resistant and methicillin-susceptible *Staphylococcus aureus*

Antibiotics	Resistance rates		p-value
MRSA, *n* = 31, *n* (%)	MSSA, *n* = 70, *n* (%)
Amikacin	3 (9.6)	2 (2.8)	0.167
Gentamicin	3 (9.6)	5 (7.1)	0.698
Tetracycline	24 (77.4)	33 (47.1)	0.005
Chloramphenicol	24 ( 77.4)	43 (61.4)	0.170
Cotrimoxazole	10 (32.3)	22 (31.4)	1.000
Ampicillin	29 (93.5)	60 (85.7)	0.335
Clindamycin	6 (19.4)	25 (35.7)	0.110
Erythromycin	8 (25.8)	15 (21.4)	0.617
Rifampicin	11 (35.5)	35 (50.0)	0.200
Streptomycin	5 (16.1)	21 (30.0)	0.217
Vancomycin	11 (35.5)	15 (21.4)	0.147

## Transparency declaration

This work was supported by the Stop Buruli initiative of the UBS Optimus foundation and the Volkswagen Foundation. The funders had no role in study design, data collection and interpretation, or the decision to submit the work for publication. Part of this work was presented at the European Congress on Tropical and Medical Health (ECTMIH) 2015 in Basel, Switzerland from 6 to 10 September 2015 with the abstract subsequently published in *Tropical Medicine and International Health* 20:68, September, 2015; the Ghana Biomedical Convention (GBC) 2015 University of Ghana, Legon from the 29 to 31 July 2015 and the 26th European Congress of Clinical Microbiology and Infectious Diseases (ECCMID), the annual yearly congress of the European Society of Clinical Microbiology and Infectious Diseases (ESCMID), in Amsterdam from 9 to 12 April 2016.

## Appendix A Supporting information

The following supplementary materials are available for this article:

**Data S1**. Antibiotic resistance genes identified

**Data S2**. Core SNP Distances

**Fig. S1**. Flowchart of study design

**Table S1**. Primer sequences

**Table S2**. *Spa* types of isolated *Staphylococcus aureus* strains

## References

[1] Robson M.C. (1997). Wound infection. A failure of wound healing caused by an imbalance of bacteria. Surg Clin North Am.

[2] Bowler P.G., Davies B.J. (1999). The microbiology of acute and chronic wounds. Wounds.

[3] Bowler P.G., Duerden B.I., Armstrong D.G. (2001). Wound microbiology and associated approaches to wound management. Clin Microbiol Rev.

[4] Brook I., Randolph J.G. (1981). Aerobic and anaerobic bacterial flora of burns in children. J Trauma.

[5] Kluytmans J., van Belkum A., Verbrugh H. (1997). Nasal carriage of *Staphylococcus aureus*: epidemiology, underlying mechanisms, and associated risks. Clin Microbiol Rev.

[6] Yeboah-Manu D., Kpeli G.S., Ruf M.T., Asan-Ampah K., Quenin-Fosu K., Owusu-Mireku E. (2013). Secondary bacterial infections of buruli ulcer lesions before and after chemotherapy with streptomycin and rifampicin. PLoS Negl Trop Dis.

[7] Clinical, Laboratory, Standards, Institute (2014). Performance standards for Antimicrobial Susceptibility Testing: Twenty fourth Informational Supplement M100–S24.

[8] Ruppitsch W., Indra A., Stoger A., Mayer B., Stadlbauer S., Wewalka G. (2006). Classifying spa types in complexes improves interpretation of typing results for methicillin-resistant *Staphylococcus aureus*. J Clin Microbiol.

[9] Votintseva A.A., Fung R., Miller R.R., Knox K., Godwin H., Wyllie D.H. (2014). Prevalence of *Staphylococcus aureus* protein A (*spa*) mutants in the community and hospitals in Oxfordshire. BMC Microbiol.

[10] AL-T F., Brunel A.S., Bouzinbi N., Corne P., Banuls A.L., Shahbazkia H.R. (2012). DNAGear–a free software for spa type identification in *Staphylococcus aureus*. BMC Res Notes.

[11] Staden R., Beal K.F., Bonfield J.K. (2000). The Staden package, 1998. Methods Mol Biol.

[12] Oliveira D.C., de Lencastre H. (2002). Multiplex PCR strategy for rapid identification of structural types and variants of the mec element in methicillin-resistant *Staphylococcus aureus*. Antimicrob Agents Chemother.

[13] Deurenberg R.H., Vink C., Driessen C., Bes M., London N., Etienne J. (2004). Rapid detection of Panton–Valentine leukocidin from clinical isolates of *Staphylococcus aureus* strains by real-time PCR. FEMS Microbiol Lett.

[14] Shopsin B., Mathema B., Alcabes P., Said-Salim B., Lina G., Matsuka A. (2003). Prevalence of agr specificity groups among *Staphylococcus aureus* strains colonizing children and their guardians. J Clin Microbiol.

[15] Price M.N., Dehal P.S., Arkin A.P. (2010). FastTree 2–approximately maximum-likelihood trees for large alignments. PLoS One.

[16] Huson D.H., Scornavacca C. (2012). Dendroscope 3: an interactive tool for rooted phylogenetic trees and networks. Syst Biol.

[17] Gastmeier P., Sohr D., Geffers C., Behnke M., Daschner F., Ruden H. (2005). Mortality risk factors with nosocomial *Staphylococcus aureus* infections in intensive care units: results from the German Nosocomial Infection Surveillance System (KISS). Infection.

[18] Egyir B., Guardabassi L., Sorum M., Nielsen S.S., Kolekang A., Frimpong E. (2014). Molecular epidemiology and antimicrobial susceptibility of clinical *Staphylococcus aureus* from healthcare institutions in Ghana. PLoS One.

[19] Breurec S., Fall C., Pouillot R., Boisier P., Brisse S., Diene-Sarr F. (2011). Epidemiology of methicillin-susceptible *Staphylococcus aureus* lineages in five major African towns: high prevalence of Panton-Valentine leukocidin genes. Clin Microbiol Infect.

[20] Schaumburg F., Kock R., Friedrich A.W., Soulanoudjingar S., Ngoa U.A., von Eiff C. (2011). Population structure of *Staphylococcus aureus* from remote African Babongo Pygmies. PLoS Negl Trop Dis.

[21] von Eiff C., Friedrich A.W., Peters G., Becker K. (2004). Prevalence of genes encoding for members of the staphylococcal leukotoxin family among clinical isolates of *Staphylococcus aureus*. Diagn Microbiol Infect Dis.

[22] Egyir B., Guardabassi L., Esson J., Nielsen S.S., Newman M.J., Addo K.K. (2014). Insights into nasal carriage of *Staphylococcus aureus* in an urban and a rural community in Ghana. PLoS One.

[23] Schaumburg F., Ngoa U.A., Kosters K., Kock R., Adegnika A.A., Kremsner P.G. (2011). Virulence factors and genotypes of *Staphylococcus aureus* from infection and carriage in Gabon. Clin Microbiol Infect.

[24] Traber K.E., Lee E., Benson S., Corrigan R., Cantera M., Shopsin B. (2008). agr function in clinical *Staphylococcus aureus* isolates. Microbiology.

[25] Kong K.F., Vuong C., Otto M. (2006). *Staphylococcus* quorum sensing in biofilm formation and infection. Int J Med Microbiol.

[26] Newman M.J., Frimpong E., Donkor E.S., Opintan J.A., Asamoah-Adu A. (2011). Resistance to antimicrobial drugs in Ghana. Infect Drug Resist.

[27] Stefani S., Chung D.R., Lindsay J.A., Friedrich A.W., Kearns A.M., Westh H. (2012). Meticillin-resistant *Staphylococcus aureus* (MRSA): global epidemiology and harmonisation of typing methods. Int J Antimicrob Agents.

[28] Petinaki E., Kontos F., Maniatis A.N. (2002). Emergence of two oxacillin-susceptible mecA-positive *Staphylococcus aureus* clones in a Greek hospital. J Antimicrob Chemother.

[29] Cuirolo A., Canigia L.F., Gardella N., Fernandez S., Gutkind G., Rosato A. (2011). Oxacillin- and cefoxitin-susceptible meticillin-resistant *Staphylococcus aureus* (MRSA). Int J Antimicrob Agents.

[30] Olayinka B.O., Olayinka A.T., Onaolapo J.A., Olurinola P.F. (2005). Pattern of Resistance to vancomycin and other antimicrobial agents in staphylococcal isolates in a university teaching hospital. Afr Clin Exp Microbiol.

[31] Onanuga A., Oyi A.R., Onoalapo J.A. (2005). Prevalence and Susceptibility pattern of methicillin resistant *Staphylococcus aureus* isolates among healthy women in Zaria, Nigeria. Afr J Biotechnol.

