# Supplementary figures and images for: Possible healthcare-associated transmission as a cause of secondary infection and population structure of Staphylococcus aureus isolates from two wound treatment centres in Ghana
Source: New Microbes New Infect. 2016 Jul 12;13:92–101. doi: 10.1016/j.nmni.2016.07.001 (PMC4983152; doi:10.1016/j.nmni.2016.07.001)

Samples

Isolates

Sampling


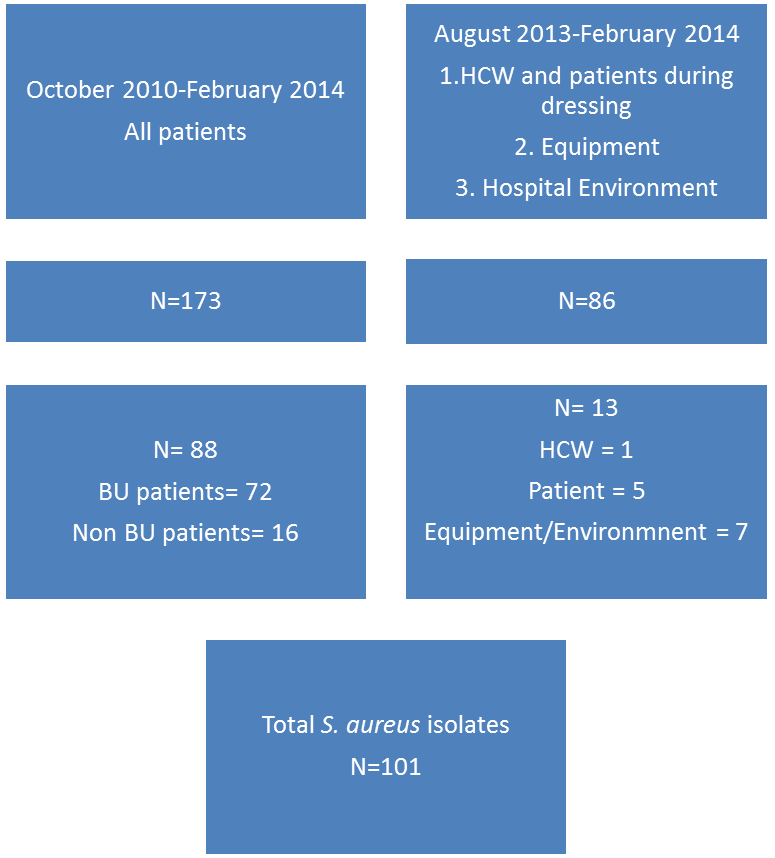


Figure S1: Flowchart of study design

Supplement: Supplementary file 3 — Fig. S1. Flowchart of study design [file mmc3.docx]
