# Supplementary material for: Possible healthcare-associated transmission as a cause of secondary infection and population structure of Staphylococcus aureus isolates from two wound treatment centres in Ghana
Source: New Microbes New Infect. 2016 Jul 12;13:92–101. doi: 10.1016/j.nmni.2016.07.001 (PMC4983152; doi:10.1016/j.nmni.2016.07.001)
Supplement: Supplementary file 4 [file mmc4.docx]

**Table S1: Primer sequences**

| Primer Name | Primer Sequence 5′--3′ |
| --- | --- |
| spa-1113F | TAAAGACGATCCTTCGGTGAGC |
| spa-1514R | CAGCAGTAGTGCCGTTTGCTT |
| spaT3-F | CAACGCAATGGTTTCATCCA |
| spa-1517R | GCTTTTGCAATGTCATTTACTG |
| mecA P4 | TCCAGATTACAACTTCACCAGG |
| mecA P7 | CCACTTCATATCTTGTAACG |
| pvl-F | GCTGGACAAAACTTCTTGGAATAT |
| pvl-R | GATAGGACACCAATAAATTCTGGATTG |
| pan agr F | ATGCACATGGTGCACATGC |
| agr 1-R | GTCACAAGTACTATAAGCTGCGAT |
| agr 2-R | GTATTACTAATTGAAAAGTGCCATAGC |
| agr 3-R | CTGTTGAAAAAGTCAACTAAAAGCTC |
| agr 4-R | CGATAATGCCGTAATACCCG |

**Table S2: Spa types of isolated *Staphylococcus aureus* strains**

| **Spa type** | **Frequency** |
| --- | --- |
| t161 | 1 |
| t 210 | 1 |
| t 460 | 1 |
| t 002 | 1 |
| t 008 | 1 |
| t 085 | 1 |
| t 1123 | 1 |
| t 127 | 2 |
| t 1458 | 1 |
| t 186 | 7 |
| t 208 | 1 |
| t 2235 | 1 |
| t 2304 | 3 |
| t 2422 | 2 |
| t 2500 | 3 |
| t 2649 | 1 |
| t 311 | 5 |
| t 314 | 2 |
| t 335 | 2 |
| t 346 | 7 |
| t 355 | 7 |
| t 448 | 2 |
| t 537 | 1 |
| t 591 | 1 |
| t 769 | 3 |
| t 786 | 3 |
| t 803 | 1 |
| t 939 | 3 |
| t 948 | 1 |
| unknown | 35 |
